# Operational evaluation of rapid diagnostic testing for Ebola Virus Disease in Guinean laboratories

**DOI:** 10.1371/journal.pone.0188047

**Published:** 2017-11-30

**Authors:** Amanda VanSteelandt, Josephine Aho, Kristyn Franklin, Jacques Likofata, Jean Baptiste Kamgang, Sakoba Keita, Lamine Koivogui, N’Faly Magassouba, Lise D. Martel, Anicet George Dahourou

**Affiliations:** 1 Epidemiology, Informatics, Surveillance and Laboratory Branch, Department of Global Health Protection, Centers for Disease Control and Prevention, Atlanta, Georgia, United States of America; 2 Canadian Field Epidemiology Program, Public Health Agency of Canada, Ottawa, Ontario, Canada; 3 Centre for Food-borne, Environmental and Zoonotic Infectious Diseases, Outbreak Management Division, Public Health Agency of Canada, Guelph, Ontario, Canada; 4 Laboratoire Provincial de Santé Publique de la Province de l'Équateur, Mbandaka, République Démocratique du Congo; 5 Agence Nationale de Sécurité Sanitaire, Conakry, Guinea; 6 Institut National de Santé Publique, Conakry, Guinea; 7 Laboratoire des Fièvres Hémorragiques en Guinée, Université Gamal Abdel Nasser, Conakry, Guinea; 8 CDC Guinea Office, Centers for Disease Control and Prevention, Conakry, Guinea; Metabiota, UNITED STATES

## Abstract

**Background:**

Rapid Diagnostic Tests (RDTs) for Ebola Virus Disease (EVD) at the point of care have the potential to increase access and acceptability of EVD testing and the speed of patient isolation and secure burials for suspect cases. A pilot program for EVD RDTs in high risk areas of Guinea was introduced in October 2015. This paper presents concordance data between EVD RDTs and PCR testing in the field as well as an assessment of the acceptability, feasibility, and quality assurance of the RDT program.

**Methods and findings:**

Concordance data were compiled from laboratory surveillance databases. The operational measures of the laboratory-based EVD RDT program were evaluated at all 34 sentinel sites in Guinea through: (1) a technical questionnaire filled by the lab technicians who performed the RDTs, (2) a checklist filled by the evaluator during the site visits, and (3) direct observation of the lab technicians performing the quality control test. Acceptability of the EVD RDT was good for technicians, patients, and families although many technicians (69.8%) expressed concern for their safety while performing the test. The feasibility of the program was good based on average technician knowledge scores (6.6 out of 8) but basic infrastructure, equipment, and supplies were lacking. There was much room for improvement in quality assurance of the program.

**Conclusions:**

The implementation of new diagnostics in weak laboratory systems requires general training in quality assurance, biosafety and communication with patients in addition to specific training for the new test. Corresponding capacity building in terms of basic equipment and a long-term commitment to transfer supervision and quality improvement to national public health staff are necessary for successful implementation.

## Introduction

The Ebola Virus Disease (EVD) outbreaks in West Africa infected more than 28,000 people and took more than 11,000 lives in Guinea, Sierra Leone, and Liberia [1]. The greatest tools for breaking the chain of transmission in these outbreaks have been (1) rapid isolation of EVD patients and (2) secure and dignified burials for victims of EVD [2]. The success of these strategies depends on the timely and reliable identification of live EVD patients and suspect deaths through laboratory testing. The gold standard for EVD diagnosis is PCR, which can be done in less than 4 hours, but requires human resources, equipment, facilities, and infrastructure unavailable in remote areas. The time to transport patients or samples to PCR capable facilities can delay results for days. Delays in test results or the need for travel can increase community resistance to public health interventions.

Rapid Diagnostic Tests (RDTs) for EVD can alleviate some of the challenges presented by PCR testing by quickly providing test results at the point of care. To expand testing services for EVD, the National Coordination for the Fight against Ebola in Guinea started a pilot program for the implementation of RDTs in the most affected regions in October 2015. The program was led by the Centers for Disease Control and Prevention (CDC) in partnership with the National coordination for the Fight against Ebola, the National Institute for Public Health, the Guinean Red Cross and International Federation of Red Cross and Red Crescent Societies, and the World Health Organization (WHO). The initial roll-out took place in the prefecture of Forécariah where active EVD transmission was present. Red Cross volunteers, who are responsible for the Secure and Dignified Burials of EVD victims, were trained to perform RDTs on the deceased and laboratory technicians at sentinel sites were trained to perform RDTs on suspect patients and patients who die in medical facilities.

Previous articles have described an initial evaluation of the pilot implementation of the EVD RDTs at 15 sites in Forécariah [3] and a baseline assessment of the use of EVD RDTs [4]. Based on lessons learned from the initial RDT implementation in Forécariah, the RDT program for patient testing rolled out more broadly in December 2015 by training (or re-training) laboratory technicians at 16 sites in Forécariah, 11 sites in Conakry, and 5 sites in the prefectures in the Forest Region that are contiguous with Liberia (Macenta, Guéckédou, N’Zérékoré, Lola, and Yomou). Two additional sites were added in the prefecture of Guéckédou in February 2016. The trainings were followed up with weekly lab visits, initial visits to reinforce the RDT training and subsequent visits by CDC or WHO epidemiologists for data collection and supportive supervision.

This paper presents the results of an evaluation of three operational measures (acceptability, feasibility, and quality assurance) conducted during the implementation of the RDT program in Guinea, as well as concordance data between EVD RDTs and PCR testing in the field.

## Methods

### Context and population

The capital city of Conakry and the prefectures of Forécariah and the Forest Region were some of the areas most affected by Ebola outbreaks. The decision to implement the RDT program in each region was based on existing EVD transmission, a high risk of transmission from a neighboring region’s outbreak, and/or a large EVD survivor population with potential viral persistence in body fluids.

The 34 sentinel labs participating in the EVD RDT program were located in Health Posts (4), Health Centers (17), Communal Medical Centers (4), and Prefectural (6) and National (3) Hospitals. Therefore their level of services, staffing, and population they served varied considerably.

### EVD Rapid Diagnostic Test

The OraQuick^®^ Ebola Rapid Antigen Test (OraSure Technologies, Inc., Bethlehem, PA) was chosen for the RDT program in Guinea based on its high manufacturer reported sensitivity (84% (95% Confidence Interval (CI): 63.92–95.46)) and specificity (98.0% (95% CI: 89.35–99.95)) for whole blood and its broad temperature tolerance for both storage (2–30°C) and testing (15–40°C) conditions [5]. The sensitivity of the test is related to the viral load in the sample, with 100% sensitivity (95% CI: 86.77–100.0%) for samples with PCR Ct range of 15 to 24 (high viral load), but 84.0% sensitivity (95% CI: 63.92–95.46%) at the full PCR Ct range of 15 to 34 (high to low viral load) [5]. OraQuick^®^ has an Emergency Use Authorization issued by the U.S. Food and Drug Administration in July 2015 [6] and was authorized for use by the Government of Guinea.

The OraQuick^®^ Ebola Rapid Antigen Test is a lateral flow, single-use immunoassay which allows qualitative detection of Ebola antigens from the whole blood of patients or saliva of corpses in 30 minutes [5]. The sample is added to the OraQuick^®^ RDT device, then the device is inserted into a vial of developer solution to facilitate the capillary flow of the specimen into the device and onto an assay strip with a Test Zone and Control Zone. As the specimen flows through the device, Ebola antigens from the specimen are bound by Ebola antibody labeled gold colorimetric reagent. If Ebola antigens are present the labeled complexes bind to the Test Zone resulting in a purple line, and if they are not present the Test Zone will remain colorless. The remaining colloidal gold continues to migrate and binds to the Control Zone resulting in a purple line to demonstrate there was adequate flow and the test was valid, regardless if the sample was positive or negative for Ebola virus. Positive results may be interpreted as soon as lines are visible at the Test and Control Zones, however negative results must be read 30 minutes after inserting the device in the developer vial to allow adequate time for migration of the sample. The intensity of the line color is not directly proportional to the amount of virus in the specimen; the test is interpreted as reactive or non-reactive.

### EVD RDT program training

More than 200 healthcare workers, laboratory technicians and laboratory trainees in Forécariah, Conakry, and the Forest Region received one-day trainings by the EVD RDT program partners. The trainings included lectures on the eligibility criteria and algorithms for the use of the EVD RDT, how to use the OraQuick^®^ EVD RDT, quality assurance, data collection and supervision tools, waste management, and communication strategies for patients and family members of the deceased as well as practice sessions with the OraQuick^®^ EVD RDTs and putting on and removing Personal Protective Equipment. Evaluations were conducted after each training to improve the content, and different instructors were involved in different regions, therefore there were some minor differences across trainings. The initial training in Forécariah in September taught that the OraQuick^®^ EVD RDT should be read at 20–30 minutes, but this guidance was updated to exactly 30 minutes in all subsequent trainings.

### Algorithms for the use of EVD RDTs

Several algorithms were used to screen for potentially unknown EVD contacts with the EVD RDTs during different time periods and in different locations. While the eligibility criteria for live patients to receive an RDT varied over place in time (i.e. matching the suspect case definition for EVD, having a febrile illness, having fever plus three other EVD symptoms), the procedures after receiving the test remained nearly the same. Patients with reactive RDTs were immediately isolated pending PCR confirmation. In the vast majority of cases, patients with non-reactive RDTs were investigated for alternative diagnoses and did not require PCR confirmation. Therefore patients with non-reactive RDTs rarely received PCR confirmation.

All deaths alerted to the surveillance system and all corpses in hospital morgues were eligible for EVD RDTs. The EVD RDT was always performed at the same time as a swab was taken for PCR confirmation. A presidential edict in place for most of the 2014–2015 Ebola outbreak required all deaths in prefectures with active transmission to receive a Secure and Dignified Burial from the Red Cross. When the edict was lifted in December 2015, a non-reactive RDT result allowed the family to proceed with a traditional burial and a reactive RDT required a Secure and Dignified Burial in communities without active transmission.

All screening algorithms required only one EVD RDT to be performed per patient or corpse, unless the first result was invalid, in which case a second RDT would be performed. If both RDTs were invalid then the algorithm proceeded to PCR testing.

### Concordance data

Concordance data for EVD RDTs and PCR were collected and monitored throughout the use of the RDTs to identify potential adverse events due to false positive or false negative results. On some occasions healthcare workers deviated from the algorithm and performed multiple EVD RDTs on one patient with discordant results; therefore number of tests was reported rather than number of persons.

Given the limited number of new EVD cases in Guinea during the implementation of this pilot project, and that PCR tests were not required for all EVD RDT tests on living patients, paired EVD RDT and PCR tests were rare for living patients. PCR testing events were reported through the National Coordination for the Fight against Ebola’s surveillance system. The case histories of these PCR testing events, investigated by field epidemiologists from CDC and WHO, would indicate if the patient had visited a sentinel lab site and the result of their RDT, if any.

Concordance data for the deceased were recorded by the Red Cross, who kept a database of RDT and PCR results, and by field epidemiologists in the Forest Region who kept line lists of RDTs performed in sentinel labs. The line lists from the Forest Region were matched with the National Coordination’s PCR testing database based on name, age, date, and location.

### Operational measures

After three months of operation, three operational measures were assessed in the laboratory-based RDT program at all 34 sentinel sites in Guinea: (1) acceptability of the test, (2) feasibility, and (3) quality assurance. The indicators and data sources used for each measure are displayed in Table 1.

**Table 1:** Operational characteristic, indicators, and data sources for evaluation.

Operational Characteristics	Indicators	Data Source		
		Technician Questionnaire	Site visit	Practical exam
Acceptability of the test	• Number of technician refusals to perform test • Number of patient refusals • Reasons given for refusals • Attitudes towards the EVD RDT (positive and negative aspects)	X		
Feasibility	• Integration into routine work (subjective) • Retention of training • Basic infrastructure, equipment, and supplies present	X	X	X
Quality Assurance	• Temperature logging • Record keeping • Stock management • Practical skills	X	X	X

Acceptability of the test includes both acceptability to patients and their families as well as acceptability to the technicians who perform the test. Feasibility involves how well technicians have retained knowledge and skills from their RDT training and their subjective opinions on how easily RDTs are integrated into their routine work. It is also related to whether or not the basic infrastructure, equipment, and supplies needed to implement the RDT program are available at the sentinel site. Quality Assurance encompasses many general quality management skills for laboratories, including temperature logging, record keeping, stock management, and quality control testing.

### Questionnaire & Site visit checklist

A technician questionnaire (S1 Annex) was designed to collect their opinions on the acceptability and feasibility of the tests as well as test their current knowledge levels. The questionnaire included qualitative and quantitative components. The questionnaire, which was written in French, was piloted at two labs in Conakry and small adjustments in wording were made based on feedback from the technicians. The final version is two pages of questions which can be completed in 15 minutes or less. In addition to demographic questions, the first page has two quantitative questions and five qualitative questions to capture the acceptability and feasibility of the EVD RDTs. The second page assesses knowledge related to quality assurance through eight quantitative questions testing current knowledge of the EVD RDTs. For example, images of four possible EVD RDT test results are given for the technicians to interpret. Questions 2, 3, and 4 were multiple choice, while questions 1, 5, 6, 7, and 8 were short answer. Only those technicians who were present during the site visit were asked to complete the survey. The responses were anonymous. Following the survey, the evaluator shared all the correct answers to the knowledge questions.

A site visit checklist (S2 Annex), completed by the evaluator, provided additional information on feasibility by examining the presence or absence of necessary materials for the RDT program, such as thermometers, timers, and log books, and whether they were used appropriately. One checklist was filled out for each sentinel site. The evaluator had a small supply of thermometers, timers, and forms to provide labs that were found lacking.

The site visits and questionnaires were conducted in late March 2016 for the 11 sentinel sites in Conakry and 16 sentinel sites in Forécariah. The March 2016 outbreak of EVD in N’Zérékoré and Macenta delayed site visits and questionnaires for the 7 sites in the Forest Region until late April to mid May 2016. A total of 87 lab technicians were surveyed, 25 from Conakry, 33 from Forécariah, and 29 from the Forest Region.

### Practical exam

Following the site visits and questionnaires, a member of the CDC Lab Team in Guinea (JL) visited 20 labs (4 in Conakry, all 7 in the Forest Region, and 9 in Forécariah) to observe lab technicians in the performance of quality control tests on EVD RDTs and provide constructive feedback. Reports from these site visits were coded for the key issues observed during test performance.

### Analysis

Quantitative data were analyzed with descriptive statistics to get the overall and regional pictures of the operational measures. Differences in knowledge scores between trainees and civil servants, those who received primary and secondary training (those who were trained by their colleague and not program partners), and across regions were assessed using the Mann-Whitney-Wilcoxon test (for two groups) and the Kruskal-Wallis test (for two-plus groups) in R statistical software version 3.2.0 [7]. Qualitative data from the survey (one to three sentence written responses) were coded for major themes by two analysts and then summarized by the percent of respondents who addressed that theme in their responses. Only codes elicited by 5% or more of respondents are presented.

### Ethics statement

The protocol for the use of EVD RDTs was approved as a non-research, program evaluation activity at CDC and authorized by the Guinean National Coordination for the Ebola Response. These data were collected as part of ongoing public health program monitoring and evaluation. Verbal consent was obtained from site supervisors and lab technicians before collecting observational data and responses to the questionnaire.

## Results

### Concordance data

The concordance between the EVD RDTs and PCR testing for both living patients and the deceased is shown in Table 2. Table 2 shows tests, rather than persons, as sometimes more than one EVD RDT was performed on the same individual with discordant results.

**Table 2:** Concordance between OraQuick^®^ Ebola Rapid Antigen Test and PCR testing for living patients and the deceased from October 2015 to April 2016 in Guinea.

**Living (blood)**					
** **	** **	**PCR**			** **
		Positive	Negative	Not Tested	Total
**RDT**	Reactive	5	*8*	0	13
	Non-reactive	**2**	22*	4117	4141
	Total	7	30	4117	4154
**Dead (swab)**					
** **		**PCR**			
** **		Positive	Negative	Not Tested	Total
**RDT**	Reactive	1	*5*	0	6
	Non-reactive	**0**	3093	52	3145
** **	Total	1	3098	52	3151

* The algorithm for RDT use in living patients did not call for a PCR test to be done for non-reactive RDTs, PCR testing was only required for reactive RDTs, except during the March 2016 outbreak when a PCR test was required for each EVD RDT on a living patient in the affected region.

In Table 2 false negative RDTs are highlighted in **bold** and false positive RDTs are highlighted in *italics*. No false negative RDTs were encountered in the deceased, but two occurred in living patients. Both false negative RDTs occurred when the RDT was used outside of the recommended protocol during the March 2016 outbreak, and both the case histories and PCR cycle threshold values suggest that the patients were at a stage of EVD with low viral load (i.e. early phase of illness or recovery phase). Both false negative RDTs were performed on known EVD contacts who should have been referred directly to PCR testing and skipped the RDT; one false negative RDT was performed by an untrained technician at a non-sentinel laboratory and the second was performed in the community (outside the laboratory) under poor conditions. None of the non-reactive RDTs that did not have a corresponding PCR test led to an outbreak event. There were eight false positive RDTs in living patients and five false positive RDTs in the deceased. The false positive rate for EVD RDTs in the deceased is 0.16% (5 of 3099 paired tests). The false negative rate for EVD RDTs in the deceased is 0% (0 of 3099 paired tests). As few of the RDTs on the living had paired tests, rates of false positives or negatives are not calculated.

### Characteristics of the sentinel laboratorians performing EVD RDTs

At the 34 sites that were surveyed, 255 laboratory workers were reported to staff the laboratories. Of these, 131 (51.4%) performed EVD RDTs. The majority (n = 76, 58.0%) of the EVD RDT users were laboratory technician trainees, and the remainders (n: 55, 41.9%) were laboratory technician civil servants. The ratio of trainees to civil servants varied by area (0.5:1 in Conakry, 1.4:1 in Forécariah and 3:1 in the Forest Region).

The questionnaire was administered to 87of the 131 laboratory workers who perform EVD RDTs; 48 (55.2%) of the respondents were trainees. Of those surveyed, 52 (59.8%) attended the primary training sessions organized by partners at the beginning of the roll-out in their region. Others received secondary training from those who had already been trained.

### Acceptability

Survey respondents described eight major positive or good features of the EVD RDT, including: (1) the ability to have a diagnosis and properly orient the patient (51.2%), (2) the rapidity of the test (30.2%), (3) the reduction of EVD spread and protection of community health (18.6%), (4) immediate medical care for patients (14.0%), (5) the facility of the test (11.6%), (6) the ability to continue surveillance and give alerts (11.6%), (7) access to PPE and disinfectants for security during the test (8.1%), and (8) the reliability of the test (8.1%). These positive features were described by respondents from all three regions, except none of the responders from Conakry listed immediate medical care for patients.

Twenty-two percent (22.0%) of respondents said there were no challenges or bad features of the EVD RDT. Other respondents described nine major challenges or bad features of the EVD RDT, including: (1) technical aspects of the test (sensitivity, specificity, reliability, etc.) (20.9%), (2) reticence or fear of violence from the patient or their entourage (19.8%), (3) the lack of financial motivation for the lab staff (12.8%), (4) difficulty in performing the test correctly (10.5%), (5) the lack of or difficulty with Personal Protective Equipment (10.5%), (6) ruptures of RDT stock (8.1%), (7) the lack of other materials or infrastructure needed to conduct the test (7.0%), (8) the lack of a designated location to don PPE and perform the test (5.8%), and (9) difficulties in the transmission of test results (5.8%). These challenges were elicited by responders from all three regions, however difficulty in performing the test correctly was not listed as a challenge in Conakry and in Forécariah neither a lack of other materials needed for the test nor difficulties transmitting test results were listed as challenges.

About half of respondents (51.2%) noted no major changes in the EVD RDT program since it began at their site. The main changes observed included less reticence from the general population (12.8%), improved performance of the test (10.5%), no more outages of EVD RDTs (5.8%), and new procedures for orienting patients (5.8%). These last two observations were only reported from for Forécariah and Conakry, the locations of the longest running sites.

The majority of respondents reported that they were concerned for their safety when performing the EVD RDT (69.8% worried, 23.3% not worried, 7.0% no response). Of those who gave reasons for their concern (n = 60), the majority stated they were concerned because despite the precautions they take there is never zero percent risk (60.0%). Others were concerned because EVD is a highly contagious and deadly disease (15.0%), because their PPE may not be correctly worn or complete (13.3%), because of reticence from the population (6.7%), or the lack of an isolation space in their facility (5.0%).

Patient refusals of an EVD RDT were encountered at least once by 14.9% of respondents, however some of these incidents may be multiple lab technicians present with the same patient. Only one lab technician reported refusing to perform one EVD RDT, but has since started to perform the test.

### Feasibility

On the ease of performing the EVD RDT: 11.6% described the RDT as easy to perform, while 10.5% listed correctly performing the test as a challenge. Furthermore, 10.5% of respondents stated that their performance of the test has improved over time.

**Fig 1:**
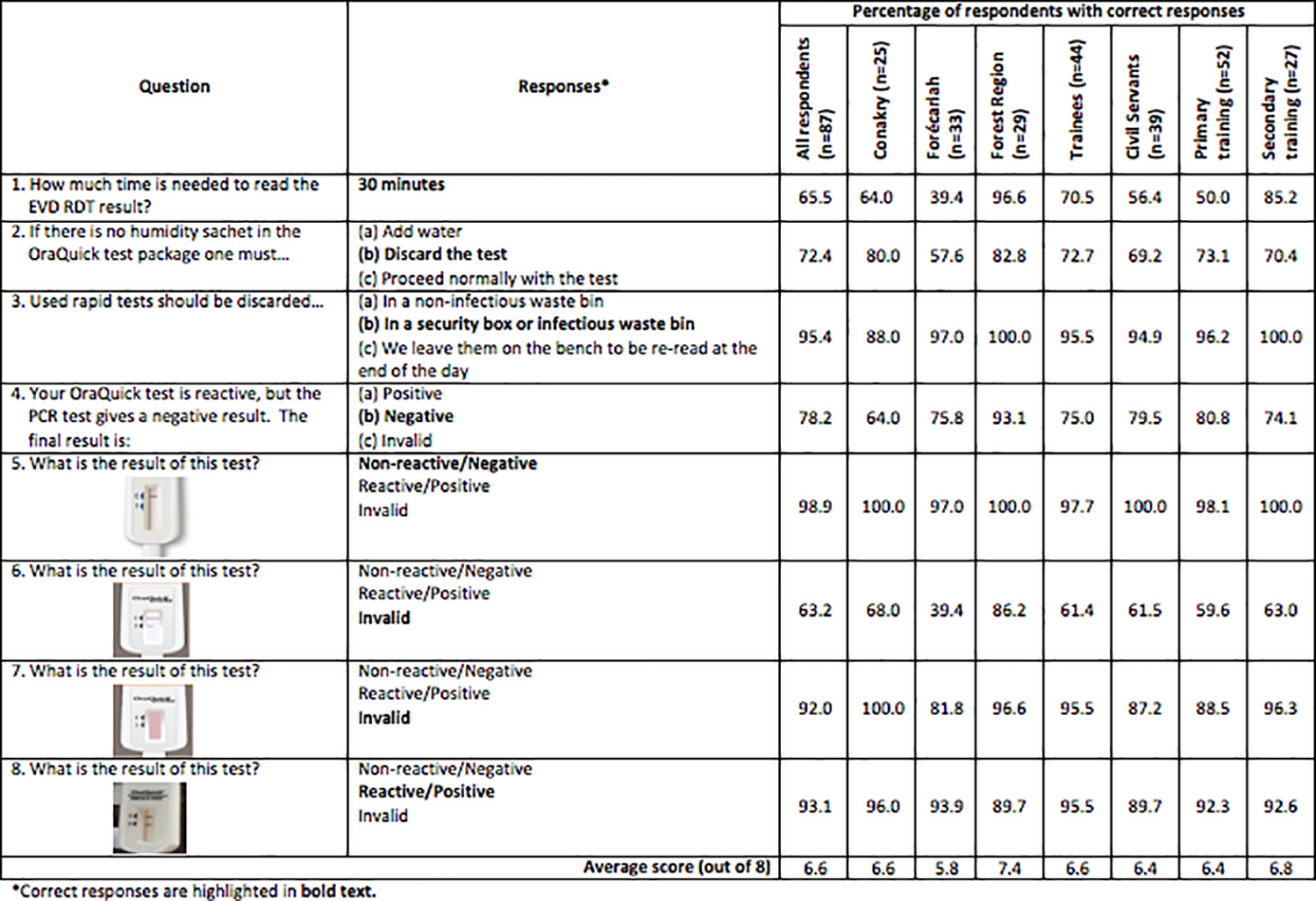
Knowledge retention of Ebola Virus Disease Rapid Diagnostic Testing. Maximum score is 8. Respondent performance is shown overall, by region, by professional status, and by training type received.

Overall, respondents performed well on the knowledge retention questions with an average score of 6.6 points of a possible 8. The average knowledge scores and the percent of respondents who answered correctly for different groups are listed in Fig 1. There was no significant difference between the knowledge scores of trainees and civil servants (W = 738.5, p-value = 0.263) or those who received primary training from partners and those who received secondary training (W = 857.5, p-value = 0.098).

There was a significant difference in the knowledge scores across regions (Kruskal-Wallis chi-squared = 28.05, df = 2, p-value < 0.001), and each region was significantly different than the other (Pairwise comparisons using the Wilcoxon test, all p-values < 0.05). Technicians in Forécariah (average score 5.8) performed the least well. Forécariah performed very poorly on Question #1 about the timing to read the RDT result, with only 39.4% of respondents answering correctly. An equal number of respondents from Forécariah (13 of 33, or 39.4%) answered 20 to 30 minutes, rather than 30 minutes, as the read time for the RDT, which agrees with the original training given in September 2015. Staff from Conakry were also challenged by this question, with only 64.0% of technicians answering correctly; while 96.6% of the Forest Region technicians answered correctly.

Respondents had little difficulty reading the reactive (93.1% correct) and non-reactive (98.9% correct) test results correctly, but had some difficulty reading the invalid test result with the incomplete “test” line (Question #6, 63.2% correct) (Fig 1). The majority of those who read the invalid test incorrectly described it as “reactive” (32.2%), which would be the more conservative response. Again, the Forest Region performed best (82.6% correct) and Forécariah (39.4% correct) the worst.

Table 3 lists by region the percentage of sites with the different elements of basic infrastructure, equipment, and supplies needed for EVD RDT quality assurance. Overall the sites were poorly equipped with the basic items needed, particularly for thermometers and up-to-date job aids. It should be noted that some surveyors counted personal cell phones as timers and others accepted only dedicated laboratory timers. A fridge, which is present at only 58.8% of the sites, is necessary for the storage of the external quality controls but not necessary for the storage of the RDTs.

**Table 3:** Percentage of laboratories with equipment for the EVD RDT program by region.

Region	% with Job Aid	% with thermometer	% with quality stock management system	% with timer	% with refrigerator	% with complete results log	% with biohazardous waste disposal	% with PPE available	% with handwashing station
Forécariah (n = 16)	37.5	0.0	25.0	93.8	56.3	0.0	100.0	100.0	100.0
Conakry (n = 11)	54.5	72.7	27.3	72.7	81.8	45.5	90.9	90.9	100.0
Forest Region (n = 7)	57.1	14.3	100.0	71.4	28.6	85.7	71.4	85.7	14.3
**Total (n = 34)**	**47.1**	**26.5**	**41.2**	**82.4**	**58.8**	**32.4**	**91.2**	**94.1**	**82.4**

The sites were better equipped for infection prevention and control with biohazardous waste disposal, PPE available, and handwashing stations in the labs. Though the Forest Region reported only 14.3% of labs having a handwashing station in the lab, there are handwashing stations available outside the lab that are shared with the rest of the facility or patients at all sites (Fig 1).

Labs that were lacking were provided with updated job aids, thermometers, timers, and RDT and PPE stock management forms during the site visit or soon afterwards. Problems with biohazardous waste management and handwashing stations were drawn to the attention of the lab staff.

### Quality assurance

Recordkeeping was very poor, with only 32.4% of sites having fully complete results log books and 41.2% having a quality stock management system. While the logbooks were not fully complete, most sites had nearly complete records. Given that only 26.5% of sites had a thermometer, temperature logging was nonexistent at most sites. Only two of the sites with thermometers kept temperature logs.

In the execution of the quality control tests with the RDTs, 70% of the sites observed–all sites in Forécariah, and about half the sites in Conakry and the Forest Region–did not use the calibrated capillary provided with the RDT kits or used the capillary incorrectly. Thirty percent (30%) of the sites observed did not have a laboratory timer or it was in poor condition and staff used personal cell phones to time the reading of the RDT. Seven sites in Forécariah (35% of sites observed) used an incorrect read time for the test. Three sites in Forécariah (15% of sites observed) were noted to have generally poor technique in the performance of the RDT. A correction and demonstration of proper technique was given to the staff on site as issues were identified.

## Discussion

Implementation of new diagnostics must take into consideration the existing level of infrastructure, equipment, and supplies in the laboratory system. The laboratory network in Guinea has very weak quality assurance and biosafety systems in place which must be built up in tandem with diagnostic capabilities. The need for quality assurance was taken into consideration during training, but these quality assurance systems will need to be reinforced and monitored over the long term. Table 4 reports the major concerns highlighted by this evaluation and the recommendations or actions taken to improve the EVD RDT program.

**Table 4:** Major concerns and recommendations and/or actions taken to improve the Ebola Virus Disease Rapid Diagnostic Testing program.

Major Concern	Recommendations and/or Actions
General public’s acceptance of the EVD RDT	Continued health promotion and education in at risk populations (communities with EVD survivors).
Weak infrastructure, equipment, and supplies for quality assurance	The EVD RDT program has defined a minimum package of equipment to be given to testing sites (including timers and thermometers). Transfer of regular quality assurance supervision to the National Institute for Public Health for long term sustainability.
Monitoring of RDT stocking temperature	Thermometers and temperature logs provided to testing sites. To supplement incomplete temperature logs, temperature sensitive stickers are used by quality assurance supervisors.
Availability of quality control testing in rural sites	New protocol was developed to transport controls in coolers for immediate use with new shipments of RDTs (no need for fridge storage).
Biosafety concerns	Infection Prevention and Control in the laboratory, including waste management and management of the Personal Protective Equipment (PPE) inventory, will be monitored during regular quality assurance visits from supervisors. PPE inventory aids were provided to RDT sites.
Adoption of updated protocols and procedures	Written procedures for version control and the dissemination of protocols to participating labs. Expectation of continuous learning communicated to lab staff.

### Acceptability

Overall the EVD RDT has a high rate of acceptability among laboratory technicians and the general public, and the acceptability has increased over time. Most of the challenges or negative aspects of the EVD RDT described by respondents can be addressed through continued health promotion and education about RDTs and support for quality lab management systems.

The mention of the technical aspects of the test (sensitivity, specificity, reliability, etc.) by 20.9% of respondents as a challenge was mostly in response to incidents of false positive or false negative tests and the need for PCR as a confirmation test. From a technical perspective, the number of false positive tests has been very low and the two false negative tests arose only when the EVD RDT was used outside of the defined algorithm. These points should be highlighted in subsequent training materials.

Request for additional financial motivation by 12.8% of laboratory workers respondents is perhaps due to the fact that incentives have been provided in the past by organizations for projects and programs related to EVD response activities and other vertical public health programs. However, the use EVD RDTs was approved as an integral function of the public health laboratories in Guinea, rather than an accessory research program. Therefore it should not require additional payment; rather it should be integrated into regular tasks. Efforts should be made to ensure the transfer of responsibility for program supervision to the Guinean government and continued technical assistance from partners.

### Feasibility

Respondents were mixed in their opinions about the ease of performing the EVD RDT but some also noted that their performance improved with practice. Objectively speaking, the EVD RDT is around the same level of difficulty as the malaria RDTs in wide use in Guinea, but the PPE requirements are more rigorous for the EVD RDT.

Overall, the lab technicians had acceptable knowledge scores, however the lack of consistency on the 30 minute read time is very concerning, especially in Forécariah. The weaker performance of the Forécariah technicians may be a result of the original training in September 2015, which recommended a 20 to 30 minute read time. The protocol has since been updated to a read time at exactly 30 minutes, but re-trainings may not have reached all the original participants. Subsequent updates to the protocol need a dissemination strategy that reaches all technicians performing the test. The non-significant difference in test scores between the technicians that received primary and secondary training suggests that re-training one focal point in each lab who would then re-train their colleagues would be a viable strategy.

Some sites that had received new equipment at the start of the EVD RDT program had lost or damaged the equipment by the time of evaluation. The lack of thermometers raises concerns about the proper storage of the RDTs. The lack of timers is particularly concerning given that an RDT non-reactive at the 30 minute read time could be read as a false positive in as little as four minutes post-read time (unpublished observation of an RDT performed on a healthy individual, CDC Guinea)–the exactness of the read time is very important. The lack of timers also becomes a biosafety concern when technicians are using their personal cell phones to time tests in the lab, especially in sites with few staff members where a colleague cannot start and stop the timer for the technician performing the RDT. Sites without refrigerators would have to receive external quality controls on ice and use them immediately upon receipt. Some sites had persistent issues with biohazardous waste disposal and the availability of PPE and handwashing stations. While many of these issues were addressed during the site visit, inventories of and improvements to the basic infrastructure, equipment, and supplies should continue on a regular basis.

### Quality assurance

The laboratory network in Guinea has little experience with quality assurance systems, thus temperature monitoring, new record keeping registers, and other lab management tasks complementary to the performance of diagnostic tests may be perceived as additional work superfluous to the use of a new diagnostic. More work is needed to integrate quality lab management systems into daily lab practice, and it should be approached on a broader basis than quality management for one diagnostic test. The difficulties identified in the use of the calibrated capillary must be addressed in future training sessions with hands-on practice.

Supervision should continue on a regular basis and should pay particular attention to any changes in protocol, such as the change in recommended read time, and adherence to the testing algorithm. Incidences of false negatives should be minimized if only trained laboratory technicians are performing the RDTs and they follow the defined screening algorithms and protocols. The algorithms used took contact history and date of symptom onset into consideration for the interpretation of the RDT result and next steps. Regular quality control testing with OraQuick^®^’s positive and negative controls allows for a check on the performance of the RDTs as well as the performance of the technicians. And continued monitoring of concordance between the EVD RDTs and RT-PCR testing allows quick identification, investigation, and response to any issues.

### Concordance data

We cannot draw conclusions about the OraQuick^®^ EVD RDT’s performance in comparison with PCR given the low prevalence of EVD during the program period. Furthermore, the use of RDT results as eligibility criteria for PCR testing among live patients prevents us from drawing conclusions about the performance of the RDT in live patients. To determine RDT performance, all RDT results should be independently compared to those of quantitative reverse transcription PCR testing, the gold-standard diagnostic assay for detecting and quantifying Ebola virus.

### Overall conclusions

The EVD RDT laboratory program is both acceptable and feasible in Guinea, but room for improvement remains, especially in quality assurance. The low percentage of false reactive OraQuick^®^ EVD RDTs among the deceased in Guinea is promising, but more data are needed on RDT performance. The cost of the RDTs and official validation of the test were not considered as part of this evaluation but might influence the long term feasibility of EVD-RDT use.

The lessons learned during the evolution of this program may benefit others who plan to implement rapid diagnostic testing during public health emergencies. The implementation of new diagnostics in weak laboratory systems requires general training in quality assurance, biosafety, and communication with patients in addition to specific training for the new test. Corresponding capacity building in terms of basic equipment and a long-term commitment to transfer supervision and quality improvement to national public health staff are necessary for successful implementation. The future impact of EVD RDTs in Guinea rests not only on strengthening these capacities but also more generally on the strengthening of communication between the laboratory and surveillance systems and the long term sustainability of the program within the Ministry of Health.

## Supporting information

S1 File Évaluation opérationnelle des Tests de Diagnostiques Rapides (TDR) pour la Maladie à Virus Ébola (MVE) dans les laboratoires Guinéens. This is a French language version of the manuscript.
(PDF)

S1 Data Database with identifying information removed in French. This database is non-public and belongs to the Ministry of Health of the Republic of Guinea. Subject to Guinean laws and regulations for the confidentiality of public health data.
(XLSX)

S2 Data Database with identifying information removed in English. This database is non-public and belongs to the Ministry of Health of the Republic of Guinea. Subject to Guinean laws and regulations for the confidentiality of public health data.
(XLSX)

S1 Annex Laboratory technician questionnaire. In French and English.
(PDF)

S2 Annex Site visit checklist. In French and English.
(PDF)
