# Supplementary material for: Operational evaluation of rapid diagnostic testing for Ebola Virus Disease in Guinean laboratories
Source: PLoS One. 2017 Nov 30;12(11):e0188047. doi: 10.1371/journal.pone.0188047 (PMC5708756; doi:10.1371/journal.pone.0188047)
Supplement: S1 File — This is a French language version of the manuscript. (PDF) [file pone.0188047.s001.pdf]

Évaluation opérationnelle des Tests de Diagnostiques Rapides pour la Maladie à Virus Ébola dans les laboratoires Guinéens

Amanda VanSteelandt[1*], Josephine Aho[2], Kristyn Franklin[3], Jacques Likofata[4], Jean Baptiste Kamgang[1], Sakoba Keita[5], Lamine Koivogui[6], N'Faly Magassouba[7], Lise D. Martel[8], and Anicet George Dahourou[1]

[1] Epidemiology, Informatics, Surveillance and Laboratory Branch, Department of Global Health Protection, Centers for Disease Control and Prevention, Atlanta, Georgia, U.S.A.

[2] Canadian Field Epidemiology Program, Public Health Agency of Canada, Ottawa, Ontario, Canada

[3] Centre for Food-borne, Environmental and Zoonotic Infectious Diseases, Outbreak Management Division, Public Health Agency of Canada, Guelph, Ontario, Canada

[4] Laboratoire Provincial de Santé Publique de la Province de l'Équateur, Mbandaka, République Démocratique du Congo

[5] Agence Nationale de Sécurité Sanitaire, Conakry, Guinea

[6] Institut National de Santé Publique, Conakry, Guinea

[7] Laboratoire des Fièvres Hémorragiques en Guinée, Université Gamal Abdel Nasser, Conakry, Guinea

[8] CDC Guinea Office, Centers for Disease Control and Prevention, Conakry, Guinea

* Auteur correspondant

E-mail: Amanda.VanSteelandt@gmail.com

Résumé

Contexte

Les Tests de Diagnostique Rapide pour la Maladie à Virus Ebola (TDR-e) au point de service ont le potentiel d'augmenter l'accès et l'acceptabilité des tests pour la MVE de même que la vitesse d'isolement des patients et les enterrements dignes et sécurisés pour les cas suspects. En octobre 2015 un programme pilote a été implémenté pour l'utilisation des TDR-e dans les zones à haute risque en Guinée. Cet article présente la concordance entre les résultats des TDR-e et PCR sur le terrain, ainsi qu'une évaluation de l'acceptabilité, de la faisabilité, et l'assurance de qualité du programme TDR-e.

Méthodes et Résultats

Les données de concordance ont été compilées des bases de données des laboratoires de surveillance. Les mesures opérationnelles du programme TDR-e basé dans les laboratoires ont été évaluées dans tous les 34 sites sentinelles en Guinée à travers : (1) un questionnaire technique rempli par les techniciens laboratoires qui effectuent les TDR-e, (2) un liste de contrôle rempli par l'évaluateur pendant les visites sur les sites, et (3) les observations directes des techniciens pendant qu'ils effectuent un test de contrôle de qualité. L'acceptabilité du TDR-e a été bonne chez les techniciens, patients et familles, malgré un grand nombre de techniciens (69.8%) inquiets pour leur sécurité pendant qu'ils effectuent le test. La faisabilité du programme est bonne basée sur le score de connaissances moyennes des techniciens (6.6 sur 8) mais les infrastructures de base, les équipements, et les provisions ont fait défaut. Il y avait beaucoup d'importantes améliorations nécessaires pour l'assurance de qualité du programme.

Conclusions

La mise en place de nouveaux tests diagnostiques dans les systèmes de laboratoires faibles nécessite une formation générale en assurance qualité, en bio-sureté et en communication avec les patients en plus d'une formation spécifique pour le nouveau test. Un renforcement des capacités en termes d'équipement de base et un engagement à long terme de transférer la supervision et l'amélioration de la qualité au personnel national de santé publique sont nécessaires pour une mise en œuvre réussie.

Introduction

Les épidémies de la Maladie à Virus Ébola (MVE) en Afrique de l'Ouest ont affecté plus que 28000 personnes et causé plus de 11000 décès en Guinée, en Sierra Leone et au Libéria [1]. Les meilleurs outils pour briser la chaîne de transmission de ces épidémies ont été (1) l'isolement rapide des patients atteints et (2) les enterrements dignes et sécurisés pour les victimes de la MVE [2]. Le succès de ces stratégies dépend de l'identification rapide et fiable des patients vivants et des personnes décédées de la MVE. La référence pour le diagnostic de la MVE est la réaction de polymérisation en chaîne (PCR), qui dure un peu moins de 4 heures, mais nécessite des ressources humaines, des équipements, des installations et une infrastructure non disponibles dans les zones isolées. Le temps requis pour transporter les patients ou les échantillons dans un endroit capable de performer les tests de PCR peut retarder de plusieurs jours l'obtention des résultats. Par ailleurs, ces retards ou la nécessité pour un cas suspect de voyager pour être testé ont un potentiel d'augmenter la réticence communautaire aux interventions de santé publique.

Les tests de diagnostic rapide (TDR) pour la MVE peuvent atténuer certains des problèmes posés par les tests de PCR en fournissant rapidement les résultats des tests au point de service. Afin d'élargir les services de dépistage de la MVE, la Coordination Nationale de Lutte contre le virus Ebola en Guinée a lancé un programme pilote de mise en œuvre des TDR dans les régions les plus touchées en octobre 2015. Le programme était dirigé par les Centres de Prévention et de Contrôle des Maladies des Etats Unis d'Amérique (CDC) en partenariat avec la Coordination Nationale de Lutte contre le virus Ebola, l'Institut National de Santé Publique, la Croix-Rouge guinéenne et la Fédération Internationale des Sociétés de la Croix-Rouge et du Croissant-Rouge et l'Organisation Mondiale de la Santé (OMS). Le déploiement initial a eu lieu dans la préfecture de Forécariah où la transmission active de la maladie à virus Ebola était présente. Les volontaires de la Croix-Rouge, responsables des enterrements dignes et sécurisés des victimes de la MVE, ont été formés pour effectuer les TDR sur le défunt et les techniciens de laboratoire des sites sentinelles ont été formés pour effectuer les TDR sur les patients suspects et les patients décédés dans les structures médicales.

Des articles précédents ont décrit l'évaluation initiale de l'implémentation pilote des TDR-e dans 15 sites à Forécariah [3] et l'évaluation de base de la mise en œuvre de l'utilisation des TDR-e [4]. Sur la base des leçons apprises de l'implantation initiale des TDR-e à Forécariah, en décembre 2015, le programme fut déployé plus largement à travers des formations (ou re-formations) offertes aux techniciens de laboratoire dans 16 sites de Forécariah, 11 sites de Conakry, et 5 sites dans les préfectures de la région forestière contiguës au Libéria (Macenta, Guéckédou, N'Zérékoré, Lola et Yomou). En février 2016, deux sites d'implémentation additionnels furent ajoutés dans la préfecture de Guéckédou. Les formations initiales furent suivies de visites de laboratoire hebdomadaires, de visites initiales pour renforcer la formation sur les TDR-e, et de visites ultérieures d'épidémiologistes de CDC ou de l'OMS pour renforcer la collecte de données et offrir une supervision formative.

Cet article présente les résultats d'une évaluation de trois mesures opérationnelles (l'acceptabilité, la faisabilité, et l'assurance de qualité) menée pendant l'implémentation du programme TDR-e en Guinée, ainsi que les données de concordance entre les résultats des TDR-e et PCR sur le terrain.

Méthodes

Contexte et Population Cible

La capitale de Conakry, les préfectures de Forécariah et la région forestière faisaient partie des régions les plus touchées par les épidémies d'Ébola. La décision de mettre en œuvre le programme TDR-e dans chacune des régions reposait sur l'existence de la transmission de la MVE, un risque élevé de transmission de l'épidémie d'une région voisine, et/ou la présence de nombreux survivants de la MVE avec persistance virale potentielle dans les fluides corporels.

Les 34 laboratoires sentinelles qui ont participé au programme de TDR-e étaient localisés dans des postes de santé (4), des centres de santé (17), des centres médicaux communaux (4), et des hôpitaux préfectoraux (6) et nationaux (3). Par conséquence, leurs niveaux de services et de ressources humaines, ainsi que la population desservie varient considérablement d'un site à l'autre.

Test Diagnostique Rapide pour la MVE

Le Test Rapide OraQuick® pour la détection de l'antigène du virus Ebola (OraSure Technologies, Inc., Bethlehem, PA) a été choisi pour le programme TDR-e en Guinée sur la base de ses hautes sensibilité (84% (Intervalle de confiance à 95% (CI): 63.92-95.46)) et spécificité (98.0% (95% CI: 89.35-99.95)) rapportées par le fabricant pour les tests effectués sur le sang total, et pour sa large tolérance de variation en température pour le stockage (2 - 30 °C) et la prise de test (15 - 40 °C) [5]. La sensibilité du test est liée à la charge virale dans l'échantillon, avec une sensibilité de 100% (IC à 95%: 86,77-100,0%) pour les échantillons ayant une valeur de cycle seuil de 15 à 24 (charge virale élevée), mais une sensibilité de 84,0% (IC à 95%: 63,92-95,46%) pour les valeurs de cycle seuil de 15 à 34 (charge virale élevée à faible) [5]. En juillet 2015, OraQuick® a reçu une Autorisation d'Utilisation en Urgence par l'U.S. Food and Drug Administration [6]. Le test a aussi été autorisé pour utilisation par le gouvernement de la Guinée.

Le Test Rapide OraQuick® pour la détection de l'antigène du virus Ebola est une méthode immuno-chromatographique à flux latéral à usage unique qui permet la détection qualitative des antigènes du virus Ebola dans le sang total des patients ou dans la salive des sujets décédés en 30 minutes [5]. L'échantillon est ajouté au dispositif du TDR OraQuick®, puis le dispositif est inséré dans un flacon de solution révélatrice pour faciliter l'écoulement capillaire de l'échantillon dans le dispositif et sur une bandelette de dosage avec une Zone de Test et une Zone de Contrôle. Lorsque l'échantillon traverse le dispositif, les antigènes d'Ébola contenus dans l'échantillon sont capturés par des anticorps anti-Ébola marqués à l'or. Si des antigènes Ebola sont présents, les complexes marqués se lient à la zone de test, ce qui donne une ligne violette, et s'ils ne sont pas présents, la zone de test restera incolore. L'or colloïdal restant continue à migrer et se lie à la zone de contrôle, ce qui donne une ligne violette démontrant que le débit était suffisant et que le test était valide, que l'échantillon soit positif ou négatif pour le virus Ebola. Les résultats positifs peuvent être interprétés dès que les lignes sont visibles dans les zones de test et de contrôle, cependant les résultats négatifs doivent être lus 30 minutes après l'insertion du dispositif dans le flacon de révélateur pour laisser le temps nécessaire à la migration de l'échantillon. L'intensité de la couleur de la ligne n'est pas directement proportionnelle à la quantité de virus dans l'échantillon; le test est interprété comme réactif ou non réactif.

Formations pour le Programme TDR-e

Plus de 200 travailleurs de la santé, techniciens et stagiaires de laboratoire de Forécariah, Conakry, et la région forestière ont reçu une formation d'une journée offerte par les partenaires du programme TDR-e. Les formations incluent des exposés sur: les critères d'éligibilité et les algorithmes d'utilisation pour les TDR-e; comment utiliser le TDR-e OraQuick®; l'assurance qualité; la collecte de données et les outils de surveillance; la gestion des déchets; et les stratégies de communication pour les patients et les membres de la famille du défunt. De plus, les participants ont bénéficié de séances pratiques pour effectuer les TDR-e OraQuick®, et porter et enlever les équipements de protection individuelle (EPIs). Une évaluation a été effectuée après chaque formation pour en améliorer le contenu. L'amélioration continue du contenu des formations et la variété des partenaires d'implémentation et des formateurs expliquent les différences mineures qui furent observées entre les formations. La formation initiale à Forécariah en septembre a enseigné la lecture du TDR-e OraQuick® en 20-30 minutes, mais une mise à jour de cette directive exigeait cette lecture à exactement 30 minutes dans toutes les formations suivantes.

Algorithmes de l'Utilisation du TDR-e

Plusieurs algorithmes ont été utilisés pour le dépistage d'éventuels contacts inconnus de la MVE avec les TDR-e pendant les différentes périodes de temps et dans les différentes régions. Même si les critères l'éligibilité des patients vivants pour être testé avec un TDR-e a varié (c'est-à-dire, se conformer à la définition de cas suspect pour la MVE, avoir une maladie fébrile, ou avoir une fièvre et trois autres symptômes de la MVE), les procédures suivies après le test étaient similaires. Les patients avec les TDR-e réactifs étaient immédiatement isolés en attendant une confirmation par PCR. Dans la grande majorité des cas, les patients avec les TDR-e non-réactifs étaient investigués pour des diagnostiques alternatifs sans confirmation par PCR. Les patients avec un TDR-e non-réactif ont donc rarement reçu un test de PCR pour Ébola.

Toutes les alertes de décès du système de surveillance et tous les corps dans les morgues des hôpitaux étaient éligibles pour les TDR-e. Le TDR-e était toujours effectué en même temps que l'écouvillonnage pour confirmation par PCR. Un décret présidentiel en place durant la période épidémique de la MVE en 2014-2015 exigeait que tous les morts dans les préfectures avec une transmission active reçoivent un Enterrement Digne et Sécurisé effectué par la Croix Rouge. Lorsque ce décret fut levé en fin décembre 2015, un résultat de TDR-e non-réactif autorisait un enterrement traditionnel dans les communautés sans transmission active tandis qu'un résultat de TDR-e réactif nécessitait un Enterrement Digne et Sécurisé.

Tous les algorithmes de dépistage exigeaient qu'un seul TDR-e soit effectué par patient ou défunt, sauf si le premier résultat était invalide, auquel cas un deuxième TDR-e serait effectué. Si les deux TDR-e étaient invalides un test de PCR était alors recommandé.

Données de Concordance

Les données de concordance pour les TDR-e et PCR étaient collectées et suivies tout au long de l'utilisation des TDR-e pour identifier potentiels évènements indésirables dus aux des faux positifs ou faux négatifs. À certaines occasions les travailleurs de santé ont dévié de l'algorithme et effectué plusieurs TDR-e sur un patient avec des résultats discordants; par conséquent, le nombre de tests est rapporté plutôt que le nombre de personnes testées.

Compte-tenu du nombre très limité de nouveaux cas d'Ébola dans le pays au moment de l'implémentation de ce projet pilote, et du fait qu'il n'était pas requis de faire des tests PCR pour tous les tests TDR-e sur les patients vivants, les patients vivants pour lesquels un TDR-e et une PCR ont pu être comparés sont peu nombreux. Néanmoins, les tests de PCR étaient tous rapportés à travers le système de surveillance de la Coordination Nationale pour la Lutte contre Ébola. L'historique de cas des PCR positifs, investigués par les épidémiologistes du terrain de CDC et de l'OMS, indiquaient si le patient a visité un laboratoire sentinelle et le résultat de leur TDR-e, si existant.

Les données de concordance pour les décès étaient rapportées par la Croix Rouge, qui ont gardé une base de données de tous leurs résultats de TDR-e et PCR, et par les épidémiologistes de terrain de la région forestière qui ont maintenu les données des TDR-e effectués dans les laboratoires sentinelles. Les données de la région forestière ont été croisées avec la base de données des tests de PCR de la Coordination Nationale en se basant sur le nom et l'âge du patient ainsi que la date et le lieu de réalisation du test.

Mesures Opérationnelles

Après trois mois de fonctionnement, trois mesures opérationnelle étaient évaluées dans le programme de TDR-e dans les 34 laboratoires sentinelles de la Guinée : (1) l'acceptabilité du test, (2) la faisabilité du test, et (3) l'assurance de la qualité. Les indicateurs et sources de données utilisées pour chaque mesure sont présentés dans tableau 1.

L'acceptabilité du test comprend l'acceptabilité pour les patients et leurs familles, ainsi que l'acceptabilité pour les techniciens qui effectuent le test. La faisabilité implique la rétention par les techniciens des connaissances et des compétences acquises lors de leur formation sur les TDR-e, de même que leur opinion sur la facilité d'intégrer les TDR-e dans leur travail de routine. La faisabilité est également liée à la présence d'infrastructure, d'équipement et des provisions de base nécessaires pour mettre en œuvre le programme TDR-e dans les sites sentinelles. L'assurance de la qualité englobe plusieurs compétences générales en gestion de la qualité pour les laboratoires, y compris l'enregistrement de la température, la tenue des dossiers, la gestion des stocks, et les tests de contrôle de la qualité.

Tableau 1. Mesures opérationnelles, indicateurs, et sources de données pour l'évaluation

**Mesures Opérationnelles**	**Indicateurs**	**Source de Données**		
		**Questionnaire de Techniciens**	**Visite du site**	**Examen pratique**
Acceptabilité du test	• Nombre de refus d'un technicien à effectuer un test<br>• Nombre de refus de patient<br>• Raisons données pour les refus<br>• Attitudes par rapport au test (aspects positifs et négatifs)	X		
Faisabilité	• Intégration dans le travail de la routine (subjective)<br>• Rétention de la formation<br>• Présence d'infrastructure, équipement et provisions de base	X	X	X
Assurance de la qualité	• Enregistrement de température<br>• Tenue des dossiers<br>• Gestion des stocks<br>• Compétences pratiques	X	X	X

Questionnaire & Liste de contrôle pour les visites de site

Un questionnaire administré aux techniciens (annexe 1) a été conçu pour recueillir leur opinion sur l'acceptabilité et la faisabilité des TDR-e, ainsi que pour tester leur niveau de connaissance actuelles. Le questionnaire comprenait une partie qualitative et une partie quantitative. Le questionnaire, écrit en français, a été mis à l'essai dans deux laboratoires de Conakry et des ajustements mineurs dans la formulation des questions ont été adoptés en fonction des commentaires des techniciens avant d'être finalisé. La version finale est divisée en deux pages de questions et peut être complétée en 15 minutes ou moins. En plus de questions démographiques, la première page contient deux questions quantitatives et cinq qualitative sur l'acceptabilité et faisabilité des TDR-e. La deuxième page évalue la connaissance liée à l'assurance de qualité avec huit questions

quantitatives pour tester les connaissances actuelles. Par exemple, des images de quatre résultats de TDR-e possibles sont présentées et le technicien doit interpréter le résultat. Les questions 2, 3 et 4 étaient à choix multiple, tandis que les questions 1, 5, 6, 7 et 8 nécessitaient une réponse courte. Seuls les techniciens présents lors de la visite du site ont été approchés pour compléter le questionnaire. Les réponses sont anonymes. Suite à l'enquête, l'évaluateur a partagé toutes les bonnes réponses aux questions de connaissances avec les personnes interrogées.

Une liste de contrôle pour les visites de sites (annexe 2), réalisée par l'évaluateur, a fourni des informations additionnelles sur la faisabilité en déterminant la présence ou l'absence de matériels nécessaires pour le programme TDR-e, tels que les thermomètres, les minuteries, les registres de laboratoire, et si elles ont été utilisées de manière appropriée. Une liste a été remplie pour chaque site sentinelle. L'évaluateur avait des thermomètres, des minuteries et des formulaires à fournir aux laboratoires qui n'en avaient pas.

Les visites de sites et l'administration de questionnaires ont eu lieu à la fin de mars 2016 pour les 11 sites sentinelles de Conakry et 16 sites sentinelles de Forécariah. À cause de l'épidémie de la MVE à N'Zérékoré et Macenta en mars 2016, les visites de site et administration de questionnaires pour les 7 sites de la région forestière furent retardés. Elles prirent place entre la fin d'avril et la mi-mai 2016. En tout, 87 techniciens de laboratoire ont répondus au questionnaire : 25 à Conakry, 33 à Forécariah et 29 dans la région forestière.

Examen Pratique

Après les visites de site et l'administration de questionnaire, un membre de l'équipe de laboratoire de CDC en Guinée (JL) a visité 20 laboratoires (4 à Conakry, tous les 7 sites dans la région forestière, et 9 à Forécariah) pour observer les techniciens de laboratoire dans leur exécution des tests de contrôle de qualité de TDR-e et leurs offrir des commentaires constructifs. Les rapports de ces visites de site ont été codés pour identifier les problèmes-clés observés pendant l'exécution du test.

Analyse

Les données quantitatives ont été analysées avec des statistiques descriptives pour obtenir des sommaires globaux et régionaux des mesures opérationnelles. Les différences en connaissance entre stagiaires et fonctionnaires, ceux qui ont reçu une formation primaire ou secondaire (qui ont été formés par leurs collègues et non par les partenaires du programme), et entre les régions ont été évaluées avec le test de Mann-Whitney-Wilcoxon (pour deux groupes) et le test de Kruskal-Wallis (pour plus de deux groupes) dans le logiciel statistique R version 3.2.0 [7]. Les données qualitatives de l'enquête (une à trois phrases par réponse écrite) ont été codées par thèmes principaux par deux analystes, et résumées par le pourcentage des répondants qui ont abordé chaque thème dans leurs réponses. Seuls les thèmes rapportés par 5% ou plus des répondants sont présentés.

Déclaration sur l'Éthique

Le protocole de ce projet a été approuvé comme une activité d'évaluation de programme (plutôt que de recherche) à CDC. Le projet a été autorisé par la Coordination Nationale pour la Lutte contre Ébola. Ces données ont été recueillies dans le cadre de la surveillance et de l'évaluation continues d'un programme de santé publique. Le consentement verbal a été obtenu par les superviseurs de site et les techniciens de laboratoire avant de recueillir des données d'observation et des réponses au questionnaire.

Résultats

Données de Concordance

La concordance entre les TDR-e et les tests de PCR pour les patients vivants et les décès est présentée sur le tableau 2. Le tableau 2 montre les tests, au lieu des personnes, parce que plus d'un TDR-e pouvait être effectué sur le même individu avec des résultats discordants.

Tableau 2. Concordance entre le Test Rapide pour les Antigènes d'Ebola OraQuick® et les tests de PCR pour les patients vivants et les décès d'octobre 2015 à avril 2016 en Guinée

**Vivants (sang)**					
		**PCR**			
		Positif	Négatif	Non Testé	Total
**TDR-e**	Réactif	5	*8*	0	13
	Non réactif	**2**	22*	4117	4141
	Total	7	30	4117	4154
**Décès (swab/écouvillon)**					
		**PCR**			
		Positif	Négatif	Non Testé	Total
**TDR-e**	Réactif	1	*5*	0	6
	Non réactif	**0**	3093	52	3145
	Total	1	3098	52	3151

* L'algorithme d'utilisation pour les TDR-e chez les patients vivants n'a pas requis un test de PCR pour les TDR-e non-réactifs, un test de PCR était seulement requis pour les TDR-e réactifs, sauf pendant la flambée en mars 2016 quand un test de PCR était requis pour chaque TDR-e avec un patient vivant dans la région affectée.

Sur tableau 2, les TDR-e faux négatifs sont en **gras** et les TDR-e faux positifs sont en *italique*. Il n'y a eu aucun TDR-e faux négatif chez les personnes décédées, mais deux se sont produits chez des patients vivants. Les deux faux négatifs ont eu lieu quand le TDR-e a été utilisé hors du protocole recommandé pendant la flambée de mars 2016, et les 2 historiques des cas et leurs valeurs de seuil de cycle de la PCR suggèrent que les patients étaient dans un stade de MVE avec une virémie faible (c'est-à-dire, phase précoce de la maladie ou phase de

convalescence). Les deux TDR-e faux négatifs étaient effectués sur les contacts de MVE connu qui auraient dû être référés directement pour un test de PCR; un TDR-e faux négatif était effectué par un technicien non-formé dans un laboratoire non-sentinelle et le deuxième était effectué dans la communauté (dehors du laboratoire) dans les conditions non adéquates. Aucun des TDR non réactifs qui n'ont pas eu de test de PCR correspondant a conduit à un événement épidémique. Il y a eu 8 TDR-e faux positifs chez les patients vivants et 5 chez les décès. Le taux de faux positifs pour les TDR-e chez les décès est de 0,16% (5 sur 3099 tests appariés). Le taux de faux négatifs chez les décès est de 0% (0 sur 3099 tests appariés). Vu que peu des TDR-e chez les vivants avaient des tests PCR appariés, les taux de faux positifs ou faux négatifs ne sont pas calculés.

Caractéristiques des laborantins des sites sentinelles qui effectuent les TDR-e

Environ 255 travailleurs de laboratoire œuvraient dans les 34 sites visités. Au total, 131 travailleurs de laboratoire (51,4%) utilisaient les TDR-e. La majorité des utilisateurs de TDR-e étaient stagiaires (n = 76, 58,0%), et le reste étaient des fonctionnaires (n = 55, 41,9%). Le ratio de stagiaires : fonctionnaires a varié par région (0.5 : 1 à Conakry, 1.4 : 1 à Forécariah et 3 : 1 dans la région forestière).

Le questionnaire a été complété par 87 (66,4%) des 131 travailleurs de laboratoire qui effectuaient des TDR-e et de ces 87 travailleurs, 48 (55,2%) étaient des stagiaires. Parmi les personnes interrogées, 52 (59,8%) ont assisté aux sessions de formation primaires organisées par les partenaires au début de la mise en place du programme TDR-e dans leur région. Les autres ont reçu une formation secondaire de ceux qui avaient déjà été formés.

Acceptabilité du TDR-e

Les répondants au questionnaire ont décrit 8 caractéristiques bonnes ou positives majeures des TDR-e: 1) la capacité d'avoir un diagnostic et d'orienter correctement le patient (51,2%), 2) la rapidité du test (30,2%), 3) la réduction de la propagation de la MVE et la protection de la santé communautaire (18,6%), 4) les soins médicaux immédiats pour les patients (14,0%), 5) la facilité du test (11,6%), 6) la possibilité de poursuivre la surveillance épidémiologique et de faire remonter les alertes (11,6%), 7) l'accès aux EPIs et désinfectants pour la sécurité pendant le test (8,1%) et 8) la fiabilité du test (8,1%). Ces caractéristiques positives ont été rapportées dans les trois régions, à l'exception qu'aucun des intervenants de Conakry n'a cité les soins médicaux immédiats pour les patients.

Vingt-deux pourcents (22,0%) des répondants ont dit qu'il n'y avait pas de défis ou mauvaises caractéristiques reliées aux TDR-e. Les autres répondants ont décrit neuf défis majeurs des TDR-e: 1) les aspects techniques du test (sensibilité, spécificité, fiabilité, etc.) (20,9%), 2) la réticence ou la peur de la violence du patient ou de leur entourage (19,8%), 3) le manque de motivation financière pour le personnel de laboratoire (12,8%), 4) difficulté d'effectuer correctement le test (10,5%), 5) l'absence ou la difficulté d'utilisation de l'équipement de protection individuelle (10,5%), 6) les ruptures du stock de TDR-e (8,1%), 7) le manque d'autres matériels ou d'infrastructure nécessaires pour réaliser le test (7,0%), 8) le manque d'un endroit désigné pour mettre les EPIs et effectuer le test (5,8%) et 9) les difficultés dans la transmission des résultats des tests (5,8%). Ces défis ont étés évoqués par les intervenants des trois régions à l'exception de la difficulté d'effectuer correctement le test (non rapporté à Conakry) et du manque d'autres matériels nécessaires pour le test ou difficultés dans la transmission des résultats des tests (non rapporté à Forécariah).

Environ la moitié des répondants (51,2%) n'a noté aucun changement majeur dans le programme TDR-e depuis qu'il a commencé à leur site. Pour ceux ayant observé des changements, les principaux changements observés

comprenaient : moins de réticences de la population (12,8%), l'amélioration de la performance du test (10,5%), moins de ruptures de stock de TDR-e (5,8%) et des nouvelles procédures pour l'orientation des patients (5,8%). Ces deux dernières observations étaient seulement pertinentes pour Forécariah et Conakry, les sites où le TDR est disponible depuis le plus longtemps.

La majorité des répondants ont indiqué qu'ils étaient inquiets pour leur sécurité lors de l'exécution du TDR-e (69,8% inquiets, 23,3% non inquiets, 7,0% pas de réponse). Parmi ceux qui ont donné des raisons pour leur préoccupation (n = 60), la majorité a déclaré qu'ils étaient préoccupés parce que, malgré les précautions qu'ils prenaient, il n'y a jamais de zéro-risque d'infection (60,0%). D'autres étaient inquiets parce que la MVE est une maladie hautement contagieuse et mortelle (15,0%), parce que leurs EPIs peuvent ne pas être correctement portés ou complets (13,3%), en raison de la réticence de la population (6,7%), ou de l'absence d'un espace d'isolement dans leur établissement (5,0%).

Le refus d'un patient d'être testé avec un TDR-e a été rencontré au moins une fois par 14,9% des répondants, mais certains de ces incidents peuvent avoir été rapportés par plusieurs techniciens de laboratoire présents avec le même patient. Un seul technicien de laboratoire a indiqué avoir refusé d'effectuer un TDR-e, mais depuis a commencé à effectuer le test.

Faisabilité des TDR-e

Sur la facilité d'exécution du TDR-e : 11,6% ont décrit le TDR-e comme facile à réaliser, tandis que 10,5% ont indiqué correctement effectuer le TDR-e comme un défi. 10,5% des répondants ont déclaré que leur performance du TDR-e s'est améliorée au fil du temps.

Dans l'ensemble, les répondants ont obtenu de bons résultats sur les questions de retention des connaissances avec un score moyen de 6,6 points sur 8 possibles. Les scores moyens et pourcentages de répondants qui ont correctement répondu pour les différents groupes sont présentés sur le tableau 3. Il n'y avait pas de différence significative entre les scores de connaissance des stagiaires et fonctionnaires ($W = 738,5$, p-value = 0.263) ou ceux qui ont reçu une formation primaire des partenaires comparés à ceux qui ont reçu une formation secondaire ($W = 857,5$, p-value = 0.098).

Il y avait une différence significative dans les scores de connaissance par région (Kruskal-Wallis chi-squared = 28,05, df = 2, p-value < 0.001), et chaque région était significativement différente des autres (comparaisons par paires à l'aide du test de Wilcoxon, toutes les valeurs $p < 0.05$). Les techniciens de Forécariah (score moyen de 5,8) ont performé le pire. Seulement 39,4% des travailleurs de Forécariah ont répondu correctement à la question 1. Un nombre égal de répondants de Forécariah (13 sur 33, soit 39,4%) ont répondu 20 à 30 minutes, plutôt que 30 minutes, pour le temps de lectures pour le TDR-e. Cette réponse concorde avec la réponse enseignée durant la formation initiale donnée en septembre 2015. Conakry a également eu difficulté avec cette question avec seulement 64,0% des techniciens répondant correctement; tandis que 96,6% des techniciens dans la région forestière ont répondu correctement.

Les répondants ont eu peu de difficulté à lire les résultats réactif et non-réactif correctement (93,1% et 98,9% respectivement), mais ont éprouvé une certaine difficulté à lire le résultat du test invalide avec trait de test incomplet (question numéro 6, 63,2% de bonnes réponses) (Tableau 3). La majorité de ceux qui ont lu le test invalide l'ont décrit incorrectement comme 'réactif' (32,2%), ce qui serait la réponse plus conservatrice. Encore une fois, la région forestière a obtenu le meilleur score (82,6%) et Forécariah le pire (39,4%).

Tableau 3. Conservation de connaissance des Tests Diagnostique Rapides pour la Maladie du Virus Ebola (score maximum = 8)

**Questions**	**Réponses***	**Pourcentage de répondants avec les réponses correctes**							
		**Tous répondants [illegible]**	**Conakry (n=25)**	**Forécariah (n=33)**	**Région Forestière [illegible]**	**Stagiaires (n=44)**	**Fonctionnaires (n=39)**	**Formation primaire [illegible]**	**Formation secondaire [illegible]**
1. Combien de temps faut-il pour lire le résultat du TDR-e	**30 minutes**	65.5	64.0	39.4	96.6	70.5	56.4	50.0	85.2
2. S'il n'y a pas de sachet pour enlever l'humidité dans le paquet du test OraQuick, il faut…	(a) Ajouter de l'eau<br>**(b) Jeter le test**<br>(c) Procéder normalement avec le test	72.4	80.0	57.6	82.8	72.7	69.2	73.1	70.4
3. On jette les tests rapides usagés…	(a) Dans une poubelle pour déchet non infectieux<br>**(b) Dans une boîte de sécurité ou une poubelle pour déchets infectieux**<br>(c) On les laisse sur la paillasse toute la journée pour relire le résultat en fin de journée	95.4	88.0	97.0	100.0	95.5	94.9	96.2	100.0
4. Votre test OraQuick est réactif, mais la PCR donne un résultat négatif, le résultat final sera….	(a) Positif<br>**(b) Négatif**<br>(c) Le résultat est invalide	78.2	64.0	75.8	93.1	75.0	79.5	80.8	74.1
5. Quel est le résultat de ce test? 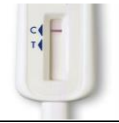	**Non-réactif/Négatif**<br>Réactif/Positif<br>Invalide	98.9	100.0	97.0	100.0	97.7	100.0	98.1	100.0
6. Quel est le résultat de ce test? 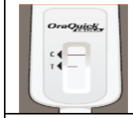	Non-réactif/Négatif<br>Réactif/Positif<br>**Invalide**	63.2	68.0	39.4	86.2	61.4	61.5	59.6	63.0
7. Quel est le résultat de ce test? 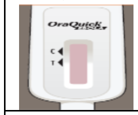	Non-réactif/Négatif<br>Réactif/Positif<br>**Invalide**	92.0	100.0	81.8	96.6	95.5	87.2	88.5	96.3
8. Quel est le résultat de ce test? 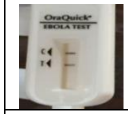	Non-réactif/Négatif<br>**Réactif/Positif**<br>Invalide	93.1	96.0	93.9	89.7	95.5	89.7	92.3	92.6
	**Score moyen (sur 8)**	6.6	6.6	5.8	7.4	6.6	6.4	6.4	6.8

*Réponses correctes souligné en **texte gras**.

Le tableau 4 présente le pourcentage des sites avec les différents éléments nécessaires pour l'assurance de qualité par région. Globalement, certains items manquaient, en particulier les thermomètres et des aide-mémoires à jour. Il faut noter que quelques administrateurs de questionnaire ont accepté les téléphones cellulaires personnels comme minuterie mais d'autres ont accepté seulement les minuteries qui appartiennent au laboratoire. Un réfrigérateur, qui était disponible dans seulement 58,8% des sites, est nécessaire pour le stockage des contrôles de qualité externes, mais pas nécessaire pour le stockage du TDR-e.

Tableau 4. Pourcentages de laboratoires avec les équipements pour le programme de TDR-e par région

**Région**	**% aide-mémoire**	**% thermomètre**	**% système de gestion de stock de qualité**	**% minuterie**	**% réfrigérateur**	**% registre de résultats complets**	**% élimination des déchets biologiques**	**% EPIs disponible**	**% station de lavage des mains**
Forécariah (n = 16)	37.5	0.0	25.0	93.8	56.3	0.0	100.0	100.0	100.0
Conakry (n = 11)	54.5	72.7	27.3	72.7	81.8	45.5	90.9	90.9	100.0
Forest Région (n = 7)	57.1	14.3	100.0	71.4	28.6	85.7	71.4	85.7	14.3
**Totale (n = 34)**	**47.1**	**26.5**	**41.2**	**82.4**	**58.8**	**32.4**	**91.2**	**94.1**	**82.4**

Les sites étaient mieux équipés pour la prévention et contrôle des infections avec l'élimination des déchets à risque biologique, la disponibilité des EPIs, et les stations de lavage de mains dans les laboratoires. Bien que la région forestière a rapporté seulement 14,3% des laboratoires ayant une station de lavage des mains dans le laboratoire, il y a des stations de lavage de mains disponibles en dehors du laboratoire qui sont partagés avec le reste de l'installation ou avec les patients dans tous les sites (tableau 3).

Les laboratoires qui manquaient d'aide-mémoires à jour, de thermomètres, de minuteries, de formulaires pour la gestion de stock des TDR-e et des EPIs en ont été donnés au cours de la visite du site ou peu après. Les problèmes liés aux stations de lavage de mains ou à la gestion des déchets biologiques ont été portés à l'attention du personnel de laboratoire.

L'Assurance de Qualité

La tenue des dossiers était très faible, avec seulement 32,4% des sites ayant les registres de résultats totalement complets et 41,2% ayant un système de gestion de stocks de qualité. Alors que les registres de résultats n'étaient pas totalement complets, la plupart des sites avaient des registres presque complets. Étant donné que seulement 26,5% des sites avait un thermomètre, l'enregistrement de la température était inexistant dans la plupart des sites. Seuls deux des sites avec des thermomètres ont enregistré les températures.

Dans l'exécution des tests de contrôle de qualité avec les TDR-e, 70% des sites observés – soit tous les sites de Forécariah et environ la moitié des sites à Conakry et dans la région forestière – n'ont pas utilisé le capillaire calibré fourni avec les kits de TDR-e ou l'ont utilisé de façon incorrecte. Trente pourcents (30%) des sites observés ne disposaient pas d'une minuterie de laboratoire où celle-ci était en mauvais état. Certains membres

du personnel utilisaient un téléphone cellulaire personnel pour chronométrer la lecture du TDR-e. Sept sites de Forécariah (35% des sites observés) ont utilisé un temps de lecture incorrecte pour le test. Trois sites de Forécariah (15% des sites observés) avaient une mauvaise technique générale dans l'exécution du TDR-e. Une correction et la démonstration de la technique appropriée fut donnée au personnel sur place lorsque un problème a été identifié.

DISCUSSION

L'implantation des nouveaux tests diagnostiques doit prendre en considération le niveau actuel de l'infrastructure, l'équipement et approvisionnement du système de laboratoire. Le réseau de laboratoire de la Guinée a des systèmes d'assurance de qualité et de biosécurité très faibles. Ceux-ci doivent être renforcés conjointement avec les capacités diagnostiques. La nécessité d'une assurance de qualité a été prise en compte lors de la formation, mais ce système devrait continuer d'être renforcé et suivis à long terme. Le tableau 5 présente les principales préoccupations mise en évidence par cette évaluation et les recommandations ou les mesures prises pour améliorer le programme TDR-e.

Tableau 5. Principales préoccupations et recommandations et/ou mesures prises pour améliorer le programme de Tests Diagnostic Rapide pour la Maladie du Virus Ébola

Préoccupation majeure	Recommandations et/ou Actions
L'acceptation de TDR-e par le public	Poursuite de la promotion de la santé et l'éducation sanitaire dans les populations à risque (les communautés avec les survivants de la MVE).
La faiblesse des infrastructures, équipements et provisions pour l'assurance de qualité	Le programme TDR-e a défini un ensemble minimum d'équipement à être accordé aux sites participants (y compris les minuteries et thermomètres). Transfert de la surveillance régulière de l'assurance de qualité à l'Institut National de la Santé Publique pour la pérennité.
Suivi de la température de stockage des TDR-e	Approvisionnement en thermomètres et registre de température aux sites participants. Pour supplémenter l'enregistrement incomplet de température, les autocollants sensibles à la température seront utilisés par les superviseurs d'assurance de qualité.
Disponibilité de tests de contrôle de qualité dans les sites ruraux	Un nouveau protocole a été conçu pour transporter les contrôles dans les glacières pour une utilisation immédiate avec chaque livraison de TDR-e (pas besoin de stockage au réfrigérateur).
Préoccupations de biosécurité et bio-sûreté	Le contrôle et prévention des infections dans les laboratoires, y compris la gestion des déchets et la gestion de l'inventaire des EPIs, seront suivis lors des visites d'assurance de qualité régulière des superviseurs. Les aides pour la gestion de l'inventaire des EPIs ont été fournis aux sites participants.
Adoption des protocoles et procédures mises à jour	Procédures écrites pour le contrôle de version et la diffusion des protocoles et procédures aux sites participants; Attente de l'apprentissage continu communiquée au personnel de laboratoire

L'acceptabilité

Globalement, le TDR-e a un taux d'acceptabilité élevé chez les techniciens de laboratoire et le grand public, et l'acceptabilité semble avoir augmenté au fil du temps. La plupart des défis ou des aspects négatifs du TDR-e décrits par les répondants peuvent être résolus par la promotion de santé, par une éducation sanitaire continue et par un soutien aux systèmes de gestion de qualité des laboratoires.

La mention des aspects techniques du test (sensibilité, spécificité, fiabilité, etc.) par 20,9% des répondants comme un défi était surtout en réponse à des incidents de faux positifs ou faux négatifs et à la nécessité du PCR comme un test de confirmation. Du point du vue technique, le nombre de faux-positifs a été très faible et les deux faux-négatifs ont surgi lorsque le TDR-e a été utilisé en dehors de l'algorithme défini. Ces points doivent être mis en évidence dans le matériel de formations futures.

La demande de motivation financière de 12,8% des répondants est peut-être liée au fait que des compensations ont été accordées dans le passé par des organisations pour les projets et programmes liés aux activités de la riposte contre la MVE et pour d'autres programmes verticaux de santé publique. Or, l'utilisation du TDR-e a été approuvée comme faisant partie intégrante du fonctionnement des laboratoires de santé publique en Guinée, plutôt qu'un programme additionnel de recherche. Il ne devrait donc pas requérir de paiement additionnel, mais plutôt être intégré aux tâches habituelles. Des efforts devraient être faits pour assurer le transfert de la responsabilité pour la supervision du programme au gouvernement et une assistance technique soutenue des partenaires.

Faisabilité

Les répondants avaient des opinions partagées sur la facilité d'exécution du TDR-e mais certains ont également remarqué que leur performance du TDR-e s'est améliorée avec la pratique. Objectivement, le TDR-e a environ le même niveau de difficulté que les TDR du paludisme qui sont largement utilisés en Guinée, mais les exigences d'EPIs sont plus rigoureuses pour le TDR-e.

Dans l'ensemble, les techniciens de laboratoire ont obtenu des scores de connaissances acceptables, mais le manque de cohérence sur le temps de lecture est très préoccupant, particulièrement à Forécariah. La performance faible des techniciens de Forécariah peut être liée à la formation initiale en septembre 2015 qui a recommandé un temps de lecture de 20 à 30 minutes. Ensuite la recommandation a changé à un temps de lecture à exactement 30 minutes, mais les formations correctives n'ont peut-être pas atteint tous les techniciens originalement formés. La différence non-significative dans les scores entre les techniciens qui ont reçu une formation primaire et ceux qui ont reçu une formation secondaire suggère que la mise à jour de la formation d'un point focal par laboratoire qui serait chargé de re-former leur collègues par la suite serait une stratégie viable.

Certains sites qui avaient reçu de nouveaux équipements au début du programme TDR-e avaient perdu ou endommagé l'équipement au moment de l'évaluation. Le manque de thermomètre soulève des inquiétudes quant à la bonne conservation du TDR-e. Le manque de minuteries est particulièrement préoccupant étant donné qu'un TDR-e non-réactif lu à 30 minutes pourrait être interprété comme un faux-positif en aussi peu que quatre minutes après le temps de lecture (observations d'un TDR-e effectué sur un individu en bonne santé non publiées, CDC Guinée). L'exactitude du temps de lecture est très importante. Le manque de minuteries devient aussi une préoccupation de biosécurité lorsque les techniciens utilisent leur téléphone cellulaire personnel pour chronométrer le temps, particulièrement dans les sites avec peu de travailleurs où un collègue ne peut pas démarrer et arrêter le chronomètre pour le technicien effectuant le TDR-e. Les sites sans réfrigérateurs devraient recevoir des contrôles de qualité externes sur glace et les utiliser immédiatement après réception. Certains sites avaient des problèmes persistants avec l'élimination des déchets biologiques et la disponibilité des EPIs et de stations de lavage de mains. Bien qu'un nombre de ces défis aient été réglés lors de la visite du site, la vérification des stocks de l'équipement, des matériaux et de l'infrastructure de base et leur amélioration devrait continuer sur une base régulière.

L'Assurance de la Qualité

Le réseau de laboratoires en Guinée a peu d'expérience avec les systèmes d'assurance de qualité. Par conséquent, la surveillance de la température, les nouveaux registres de tenue de dossiers, et d'autres tâches de gestion de laboratoire complémentaire à la réalisation de tests de diagnostic peuvent être perçus comme un travail supplémentaire lié au TDR-e, plutôt que de procédures standards de routine. Des efforts devraient être faits pour encourager l'intégration de ces mesures d'assurance de qualité dans les procédures standards de routine, et il devrait être abordé sur une base plus large que la gestion de la qualité pour un seul test diagnostique. Les difficultés identifiées dans l'utilisation du capillaire calibré doivent être abordées dans les futures sessions de formation avec une session de pratique.

La supervision devrait se poursuivre sur une base régulière et devrait accorder une attention particulière à tout changement de protocole, telles que le changement dans le temps de lecture recommandée et à le respect de l'algorithme de test. Les incidences de faux négatifs devraient être minimisées si seulement les techniciens de laboratoires formés effectuent les TDR-e et ils suivent les algorithmes et protocoles de dépistages définis. L'histoire du contact et la date d'apparition des symptômes ont fait partie de l'interprétation du résultat du TDR-e et les prochaines étapes dans les algorithmes. Les tests de contrôle de qualité réguliers avec les contrôles positifs et négatifs de l'OraQuick® permettent une vérification de la performance des TDR-e aussi que la performance des techniciens. Et le suivi continu de la concordance entre les TDR-e et RT-PCR permet l'identification, l'investigation et riposte rapide à tous les problèmes.

Données de Concordance

Nous ne pouvons pas conclure sur la performance du TDR-e OraQuick® en comparaison avec PCR vu le bas taux de prévalence de la MVE pendant le période du programme. En outre, l'utilisation des résultats des TDR-e comme critère d'éligibilité pour la PCR dans les patients vivants nous empêche de conclure sur la performance du TDR-e dans les patients vivants. Pour déterminer la performance du TDR, tous les résultats du TDR devraient être comparés indépendamment avec les tests de PCR quantitative en temps réel, le standard d'excellence pour détecter et quantifié le virus Ébola.

Conclusions Générales

Le programme TDR-e dans les laboratoires est à la fois acceptable et réalisable en Guinée, mais peut bénéficier encore d'amélioration, particulièrement dans l'assurance de qualité. Le bas pourcentage de TDR-e OraQuick® faux réactifs avec les décès en Guinée est promettant, mais plus de données sont nécessaires pour évaluer la performance du TDR. Le coût des TDR-e propres et la validation formelle du test n'ont pas été pris en considération dans cette étude et pourrait influencer la faisabilité de l'utilisation du TDR-e à long terme.

Les leçons apprises pendant l'évolution du programme peuvent bénéficier à d'autres programmes d'implantation de tests diagnostiques rapides pendant une urgence sanitaire. La mise en place de nouveaux tests diagnostiques dans les systèmes de laboratoires faibles devrait inclure des éléments généraux de formation en assurance de qualité, en bio-sureté et en communication avec les patients en addition à ceux plus spécifiques à ces nouveaux tests. Le développement correspondant de capacités en termes d'équipements de base et d'engagement à long-terme pour transférer les responsabilités de supervision et d'amélioration de la qualité au personnel national de la santé publique sont nécessaires pour une implémentation fructueuse. L'impact futur des TRD-e en Guinée repose non seulement sur le renforcement de ces capacités mais aussi plus

généralement sur le renforcement de la communication d'information entre les systèmes de laboratoire et de surveillance et sur la pérennisation soutenable à long terme du programme au sein du Ministère de la Santé.

REMERCIEMENTS : Merci aux travailleurs de la santé et aux techniciens laboratoires qui ont participé au programme pilote, et à nos partenaires de l'Organisation Mondiale de la Santé (particulièrement Koumpingnin Yacouba Nebie), la Fédération Internationale de la Croix Rouge et de la Croix Rouge Guinéenne. Jean-Paul Moke Six, Ramatoulie Jallow, Patrick Mavungu, Giulia Earle-Richardson, et François Mbuyu Kata ont joué un rôle majeur dans la collecte des données.

REFERENCES

1. World Health Organization situation report – 10 June 2016. Geneva, Switzerland: World Health Organization, 2016. http://apps.who.int/ebola/ebola-situation-reports
2. Lindblade KA, Nyenswah T, Keita S, Diallo B, Kateh F, Amoah A, et al. Secondary infections with Ebola virus in rural communities, Liberia and Guinea, 2015-2015. Emerg Infect Dis. 2016. http://dx.doi.org/10.3201/eid2209.160416
3. Jean Louis F, Huang J, Yacouba N, Koivogui L, Jayaraman G, Abiola N, et al. Implementation of Ebola rapid diagnostic tests in 15 health facilities in Forécariah, Guinea. African Journal of Laboratory Medicine. 2017; 6 (1). doi:10.4102/ajlm.v6i1.484
4. Huang JY, Jean Louis F, Dixon MG, Sefu M, Kightlinger L, Martel LD, et al. Notes from the field: Baseline Assessment of the Use of Rapid Diagnostic Tests – Forécariah, Guinea, October – November 2015. MMWR. 2016; 65 (12): 328-329.
5. OraSure Technologies, Inc. OraQuick® Ebola Rapid Antigen Test: Instructions for Use – Whole Blood. Bethlehem, PA, U.S.A. 2016. Available at: http://www.orasure.com/products-infectious/products-infectious-oraquick-ebola.asp
6. The U.S. Food and Drug Administration Emergency Use Authorizations. Available at: https://www.fda.gov/EmergencyPreparedness/Counterterrorism/MedicalCountermeasures/MCMLegalRegulatoryandPolicyFramework/ucm182568.htm#ebola
7. R Core Team. R: A language and environment for statistical computing. R Foundation for Statistical Computing, Vienna, Austria; 2015. URL http://www.R-project.org/.
