# Supplementary material for: Operational evaluation of rapid diagnostic testing for Ebola Virus Disease in Guinean laboratories
Source: PLoS One. 2017 Nov 30;12(11):e0188047. doi: 10.1371/journal.pone.0188047 (PMC5708756; doi:10.1371/journal.pone.0188047)
Supplement: S1 Annex — In French and English. (PDF) [file pone.0188047.s004.pdf]

Évaluation du Programme Pilote avec les TDR-e

Nom du laboratoire : ______________________________

Profession : _______________________________________ Stagiaire? **Oui** **Non**

Date et lieu de formation : _________________________ Formation de CDC? **Oui** **Non**

Quels sont les aspects positifs (bons côtés) de l'utilisation du TDR-e?

____

____

____

Quels sont les défis (difficultés, mauvais côtés) dans l'utilisation du TDR-e?

____

____

____

Est-ce que les bons côtés et les mauvais côtés des TDR-e ont changé depuis que vous avez commencé à utiliser le test? Si oui, comment ont-ils changé?

____

____

Êtes-vous inquiet au sujet de votre sécurité pendant que vous faites le TDR-e? **Oui** **Non**

Si Oui, pourquoi? ____

<u>Combien de fois</u> vous n'avez pas fait un TDR-e recommandé par le clinicien parce que:

(a) Le patient a refusé le TDR-e : ______ (b) Manque de TDR-e : _____

(c) Manque d'EPIs : ______ (d) Autre raison : _____

Expliquer les autres raisons :

____

Évaluation du Programme Pilote avec les TDR-e

L'objectif des questions suivantes est d'évaluer les formations sur les TDR-e. Veuillez-répondre au mieux de votre connaissance; nous souhaitons répondre aux inquiétudes ou questions sur les procédures du test au besoin après ce questionnaire.

1. Combien de temps faut-il pour lire le résultat du TDR-e? _______________

2. S'il n'y a pas de sachet pour éviter l'humidité dans le paquet du test Oraquick, il faut :
   a. Ajouter de l'eau
   b. Jeter le test
   c. Procéder normalement avec le test

3. On jette les tests rapides usagés :
   a. Dans une poubelle pour déchets non infectieux
   b. Dans une boîte de sécurité ou une poubelle pour déchets infectieux
   c. On les laisse sur la paillasse toute la journée pour relire le résultat en fin de journée

4. Votre test Oraquick est réactif, mais la PCR donne un résultat négatif, le résultat final sera :
   a. Positif
   b. Négatif
   c. Le résultat est invalide

5. Selon vous, quels sont les résultats des TDR-e suivants:

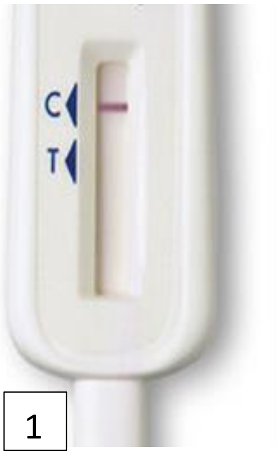

1

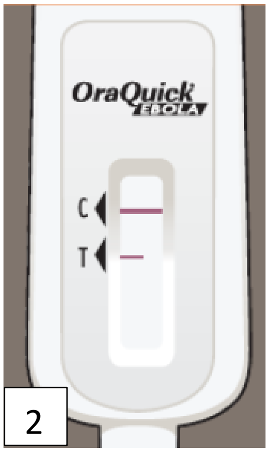

2

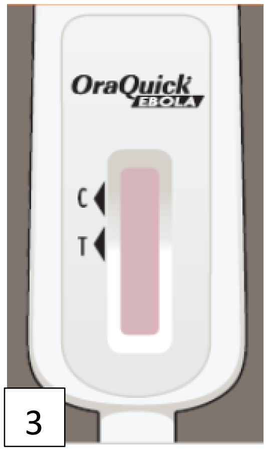

3

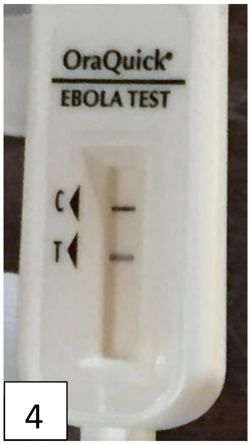

4

1	2	3	4

Evaluation of Ebola RDT Pilot Program

Laboratory name : ______________________________

Profession : ______________________________________ Trainee? **Yes** **No**

Date and place of training : _________________________ CDC training? **Yes** **No**

What are the positive aspects (good sides) of using the Ebola RDT?

____

____

____

What are the challenges (difficulties, bad sides) of using the Ebola RDT?

____

____

____

Have the good sides and bad sides of the Ebola RDT changed since you started using the test? If yes, how have they changed?

____

____

Are you worried about your safety while you perform the Ebola RDT? **Yes** **No**

If Yes, why? ____

How many times have you not done an Ebola RDT recommended by a clinician because:

(a) The patient refused the Ebola RDT: ______ (b) Lack of Ebola RDT: _____

(c) Lack of PPE: ______ (d) Other reason : _____

Explain the other reasons:

____

Evaluation of Ebola RDT Pilot Program

The objective of the following questions is to evaluate the trainings on the Ebola RDT. Please respond to the best of your knowledge; we wish to answer any concerns or questions about test procedures as needed after this questionnaire.

1. How much time is needed to read the Ebola RDT test result? ________________

2. If there is no packet to prevent humidity in the Oraquick kit, you must :
   a. Add water
   b. Throw away the test
   c. Proceed normally with the test

3. We throw used rapid tests :
   a. In a bin for non-infectious waste
   b. In a security box or bin for infectious waste
   c. We leave them on the counter all day to re-read the result at the end of the day

4. If your Oraquick test is reactive, but the PCR test gives a negative result, the final result would be:
   a. Positive
   b. Negative
   c. The result is invalid

5. According to you, what are the results of the following Ebola RDTs:

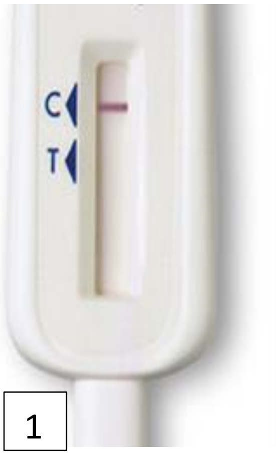

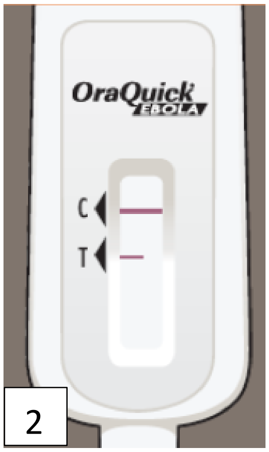

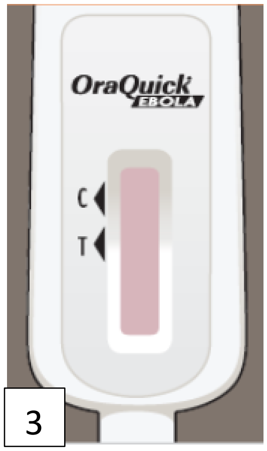

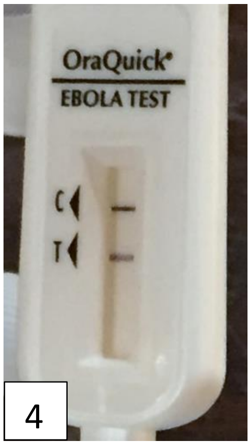

1	2	3	4
