# Supplementary material for: Operational evaluation of rapid diagnostic testing for Ebola Virus Disease in Guinean laboratories
Source: PLoS One. 2017 Nov 30;12(11):e0188047. doi: 10.1371/journal.pone.0188047 (PMC5708756; doi:10.1371/journal.pone.0188047)
Supplement: S2 Annex — In French and English. (PDF) [file pone.0188047.s005.pdf]

**Évaluation du Programme Pilote avec les TDR-e**

**CHECKLIST POUR VISITE DE LABORATOIRE**

Date: ____________

Nom du laboratoire : ____________________________

| ITEMS | OUI | NON |
|---|---|---|
| 1. POS ou aides mémoire sont disponibles, visibles et à jour | | |
| 2. Thermomètre dans le site de stockage pour les TDR-e | | |
| 3. Les kits sont stockés à la bonne température (2-30 C) | | |
| 4. Suivi des stocks comprend le numéro de lot et la date d'expiration | | |
| 5. Thermomètre dans le site ou les TDR-e sont effectues | | |
| 6. Le site pour faire le test à la bonne température (15-40 C) | | |
| 7. Log de températures de labo est rempli chaque jour | | |
| 8. Chronomètre au site de test (cellulaire okay) | | |
| 9. Frigo (de 2-8 C) au laboratoire | | |
| 10. Contrôles au laboratoire | | |
| 11. Registre de contrôles faites au laboratoire | | |
| 12. L'écriture est lisible sur les registres du labo | | |
| 13. Les registres sont remplis au complet | | |
| 14. Poubelles pour les déchets dangereux et non dangereux dans le laboratoire | | |
| 15. EPIs (gants, blouse, protection pour le visage) sont disponibles | | |
| 16. Installation de lavage des mains est disponible | | |
| 17. Disponibilité d'un téléphone pour envoyer des données | | |

| | # personnes | |
|---|---|---|
| | Titulaires | Stagiaires |
| Personnes qui ont rempli un questionnaire | | |
| Total de personnes qui font les TDR-e | | |
| Total de personnes qui travaillent dans ce labo | | |

Notes :

**Evaluation of Ebola RDT Pilot Program**

**CHECKLIST FOR LABORATORY VISIT**

Date: ____________

Laboratory name : ____________________________

| ITEMS | YES | NO |
|---|---|---|
| 1. SOP or job aids are available, visible and up-to-date | | |
| 2. Thermometer in the storage site for the Ebola RDTs | | |
| 3. The kits are stored at the correct temperature (2-30 C) | | |
| 4. Inventory tracking includes the lot number and expiration date | | |
| 5. Thermometer in the site where Ebola RDTs are performed | | |
| 6. Site for performing the Ebola RDT is at the correct temperature (15-40 C) | | |
| 7. The laboratory temperature log is filled each day | | |
| 8. Timer at the testing site (cellphone okay) | | |
| 9. Refrigerator (at 2-8 C) in the laboratory | | |
| 10. Controls at the laboratory | | |
| 11. Register of control tests done at the laboratory | | |
| 12. Writing is legible on the laboratory registers | | |
| 13. The registers are completed in full | | |
| 14. Waste bins for hazardous and non-hazardous waste in the laboratory | | |
| 15. PPE (gloves, gown, face protection) are available | | |
| 16. Handwashing station is available | | |
| 17. A telephone for sending data is available | | |

| | # persons | |
|---|---|---|
| | Functionaries | Trainees |
| Persons who completed a questionnaire | | |
| Total persons who perform Ebola RDTs | | |
| Total persons who work in this laboratory | | |

Notes :
